# Metabolomics Profiling and Diagnosis Biomarkers Searching for Drug-Induced Liver Injury Implicated to *Polygonum multiflorum*: A Cross-Sectional Cohort Study

**DOI:** 10.3389/fmed.2020.592434

**Published:** 2020-11-30

**Authors:** Ying Huang, Xu Zhao, Zi-teng Zhang, Shuai-shuai Chen, Shan-shan Li, Zhuo Shi, Jing Jing, Ang Huang, Yu-ming Guo, Zhao-fang Bai, Zheng-sheng Zou, Xiao-he Xiao, Jia-bo Wang, Ming Niu

**Affiliations:** ^1^College of Pharmacy, Hunan University of Chinese Medicine, Changsha, China; ^2^China Military Institute of Chinese Medicine, Fifth Medical Center of Chinese PLA General Hospital, Beijing, China; ^3^Center for Non-Infectious Liver Disease, Fifth Medical Center of Chinese PLA General Hospital, Beijing, China; ^4^Department of Poisoning Treatment, Fifth Medical Center of Chinese PLA General Hospital, Beijing, China

**Keywords:** drug-induced liver injury, metabolomics, autoimmune hepatitis, hepatitis B, *Polygonum multiflorum* Thunb 3

## Abstract

**Aim:** The diagnosis of drug-induced liver injury (DILI) remains a challenge and the cases of *Polygonum multiflorum* Thunb. (PM) induced DILI (PM-DILI) have received much attention This study aimed to identify a simple and high-efficiency approach to PM-DILI diagnosis via metabolomics analysis.

**Methods:** Plasma metabolites in 13 PM-DILI patients were profiled by liquid chromatography along with high-resolution mass spectrometry. Meanwhile, the metabolic characteristics of the PM-DILI were compared with that of autoimmune hepatitis (AIH), hepatitis B (HBV), and healthy volunteers.

**Results:** Twenty-four metabolites were identified to present significantly different levels in PM-DILI patients compared with HBV and AIH groups. These metabolites were enriched into glucose, amino acids, and sphingolipids metabolisms. Among these essential metabolites, the ratios of P-cresol sulfate vs. phenylalanine and inosine vs. bilirubin were further selected using a stepwise decision tree to construct a classification model in order to differentiate PM-DILI from HBV and AIH. The model was highly effective with sensitivity of 92.3% and specificity of 88.9%.

**Conclusions:** This study presents an integrated view of the metabolic features of PM-DILI induced by herbal medicine, and the four-metabolite decision tree technique imparts a potent tool in clinical diagnosis.

## Introduction

Drug-induced liver injury (DILI) includes an assorted array of symptoms caused by exposure to synthetic or natural drugs, and the incidence rate is 1–10 in 100,000, approximately. DILI is one of the major reasons for the effects of withdrawal of drugs (1). A sustained growth in the number of patients with DILIs has been recorded in recent years, particularly due to the rapid expansion of the use of natural herbal medicines, including traditional Chinese medicines (TCM) and herbal healthcare products (2–4). DILI patients can deteriorate due to acute liver failure (ALF), and more than half of ALF patients have been reported to be attributed to DILI (5). The early diagnosis of DILI is crucial to ameliorate the therapeutic outcome.

However, the diagnosis of DILI is quite challenging due to the lack of specific and reliable biomarkers (6), and largely depending on excluding diagnosis. Moreover, the diagnostic approach is complex and time-consuming (7). Recently, *Tomoyoshi* et al. reported that γ-Glu-citrulline and ALT have the potential to differentiate DILI from other liver diseases, including hepatitis B (HBV), and hepatitis C (HCV) (8). Furthermore, in our previous study, nine biomarkers obtained by means of ^1^H NMR were employed in the differential diagnosis of AIH from other diseases that are commonly confused with AIH, including DILI (9). However, the efficiency of these biomarkers in the differentiation of DILI and viral hepatitis (VH) is unclear. Therefore, we need to focus on identifying more specific biomarkers of DILI.

Metabolomics focuses on the global metabolic changes that occur in response to disturbances in living systems, and has emerged as a powerful tool for disease diagnosis and pathogenic investigations (10). In the present study, the metabolites DILI patients were examined by metabolomics involving the use of liquid chromatography mass spectrometry (LC/MS). Due to the rise in number of cases of *Polygonum multiflorum* Thunb. (PM) induced DILI (PM-DILI) which has received much attention in Asian countries (e.g., China, Japan, and Korea), as well as European and North American countries (11, 12). PM-DILI was explored in this study. We identified a highly specific set of metabolites (i.e., P-cresol sulfate, phenylalanine, inosine, and bilirubin) that were able to effectively differentiate PM-DILI patients from patients with other applicable diseases and healthy controls.

## Materials and Methods

### Patients

A total of 58 plasma samples were obtained from patients in the fifth Medical center of Chinese PLA general hospital. Thirteen PM-DILI patients, 12 AIH patients, 24 HBV patients, and 9 healthy controls were recruited after they provided informed consent; the study protocol was approved by the Medical Ethics Committee of the Fifth Medical Center of Chinese PLA General Hospital. The diagnoses of all patients were made according to international codified criteria (7, 13, 14). The plasma samples of AIH and HBV patients were collected during acute exacerbation while hospitalized. The clinical baseline characteristics, including gender, age, and the main parameters of the liver markers, are summarized in Table 1.

**Table 1:** Clinical baseline characteristics of all participants^a^.

**Variable (Mean ± SD)**	**AIH**	**CON**	**DILI**	**HBV**
Gender (F/M)^b^	9/3	2/7	6/7	2/22
Age (y)	52.8 ± 11.5	51 ± 16	45.8 ± 13	45.0 ± 9.6
IgG (g/L)	35.3 ± 3.7	9.7 ± 2.2	32.2 ± 5.4	29.5 ± 4.5
IgM (g/L)	33.6 ± 5.4	1.8 ± 1.2	28.8 ± 17.1	30.1 ± 10.7
Bilirubin (μmol/L)	26.5 ± 17.2	12 ± 7	85.8 ± 90.5	252.0 ± 127.5
ALT (U/L)	55.8 ± 24.4	31 ± 11	372.2 ± 424.5	76.6 ± 55.3
AST (U/L)	62.9 ± 26.9	27 ± 10	210.2 ± 202.3	111.0 ± 69.1
ALP (U/L)	183.1 ± 94.7	77 ± 40	127.9 ± 48.7	134.6 ± 35.7

a *SD, standard deviation; AIH, autoimmune hepatitis; CON, health controls; DILI, Drug-induced liver injuries; HBV, hepatitis; IgG, immunoglobulin G; IgM, immunoglobulin M; ALT, alanine aminotransferase; AST, aspartate aminotransferase; ALP, alkaline phosphatase*.

b *F for female and M for male*.

### Sample Preparation

Pre-prandial venous blood samples were collected in the morning using lithium heparin tubes (BD Vacutainer; 6 mL; Becton, Dickinson and Company, Franklin Lakes, NJ, USA), and the plasma was collected by centrifugation at 1,000 g at 4°C for 15 min. The plasma samples were immediately stored at −80°C until they were used for the metabolomics analysis. Prior to the LC/MS analysis, 600 μl of acetonitrile was added to 200 μl of plasma, which was thawed at room temperature, for sample purification. The samples were then centrifuged at 10,000 g for 10 min at 4°C. Subsequently, the supernatants were transferred into sample vials.

### UHPLC/MS-QTOF Measurement

Four-microliter samples were injected and separated using an Infinity 1290 UHPLC system (Agilent Technologies, SA, USA), ZORBAX SB 300 C18 column (100 × 2.1 mm with a 1.8-μm particle size, Agilent Technologies, SA, USA). The column temperature was maintained at 35°C. The system was operated at a flow rate of 0.3 mL/min with solvent A (water) and solvent B (acetonitrile), and 0.1% of formic acid was also added for the positive mode. The gradient elution program was as follows: 5.00% B for min 0–1; 5%−40% B for min 1–9; 40–90% B for min 9–19; 90–100% B for min 19–21; and 100% A for min 21–25. The total run time was 30 min for each analysis. The mass data were acquired with an Agilent 6550A Q-TOF mass spectrometer (Agilent Technologies, SA, USA) in the full scan mode (80–1,200 m/z) in both the positive and negative ion modes using an Agilent Jet Stream ESI source. In the positive mode, the capillary voltage was set to 4,000 V with a nozzle voltage of 500 V. In the negative mode, the capillary voltage was set to −3,000 V with a nozzle voltage of −500 V. The other source parameters were as follows: the nebulizer was set to 45 psig; the drying gas temperature was maintained at 225°C; the flow rate was 11 L/min; and the voltages of the fragmentor, skimmer 1, and octupole RF peak were 230, 0, and 750 V, respectively. During the analysis, two groups of reference masses of 121.0509 m/z (purine; [C_5_H_4_N_4_ + H]^+^) and 922.0098 m/z (HP-0921; [C_18_H_18_O_6_N_3_P_3_F_24_ + H]^+^) in positive mode and 112.9885 m/z (ammonium trifluoroacetate [C_2_H_4_O_2_NF_3_ - NH_4_]^−^) and 1,033.9881 m/z (HP-0921 + ammonium trifluoroacetate [C_20_H_22_O_8_N_4_P_3_F_27_ - NH_4_]^−^) in negative mode, were continuously injected to obtain high-accuracy mass correction. To ensure the stability and repeatability of the LC-MS systems, the QC sample was obtained from 10 μl of each sample and then analyzed together with the other samples. Five replicates of the QC sample were performed on the system before the sample sequence. The QC samples were also inserted, and every five samples were analyzed.

### Data Processing and Statistical Analysis

The peak alignment and data filtering were processed using Mass Profinder (version B.06.00, Agilent Technologies, SA, USA). For the molecular feature extraction, up to 2,000 compounds with peak height above 300 counts were extracted. GeneSpring (version 13.1.1, Agilent Technologies, SA, USA) was used for the normalization and statistical analysis. Only variables that were present in 70% of at least one group were included in the analysis, to reduce noise. The normalized data was analyzed using the Wilcoxon Mann-Whitney Test with *p* < 0.05 and a fold change > 2 set as the level of statistical significance. SIMCA 13.0 (Umetrics, Umeå, Sweden) was used for the multivariate analysis, principal component analysis (PCA) and orthogonal projection to latent structures discriminate analysis (OPLS-DA) were applied with Pareto scaling. Finally, the metabolites with greater variable importance in the projection value (VIP > 1) and correlation coefficient value (|Pcorr| > 0.5) in the OPLS-DA analysis were considered statistically significant.

### Biomarker Identification

The compounds that exhibited significant changes were selected as the candidate biomarkers and identified using the METLIN database (http://metlin.scripps.edu/). The pathway analysis of the potential biomarkers was carried out with MetaboAnalyst 3.0 (http://www.metaboanalyst.ca/) based on the Kyoto Encyclopedia of Genes and Genomes (KEGG) pathways library for humans (http://www.genome.jp/kegg/). The differences were considered significant when the test *p* value was below 0.05.

### Decision Tree Learning for the PM-DILI Diagnosis

The classification and regression tree analysis (CRT) was calculated with IBM SPSS 22.0 for Windows, and the Gini algorithm together with a 10-fold cross-validation was used to determine the best split for each node. Receiver operating characteristic (ROC) testing was performed to further evaluate the performances of the established models in the diagnoses of the different patients. The diagnostic values were assessed using the sensitivity, specificity, and area under the curve (AUC).

## Results

### Plasma Metabolomic Study of the Patients

The representative UHPLC-MS base peak ion (BPI) current chromatograms in the positive and negative ion modes for the human sera from each group, were visually compared. Using the optimized analysis protocol, such as peak alignment and normalization, we obtained 3,995 molecular features.

### Pattern Recognition Analysis and Biomarker Screening

**Figure 1:**
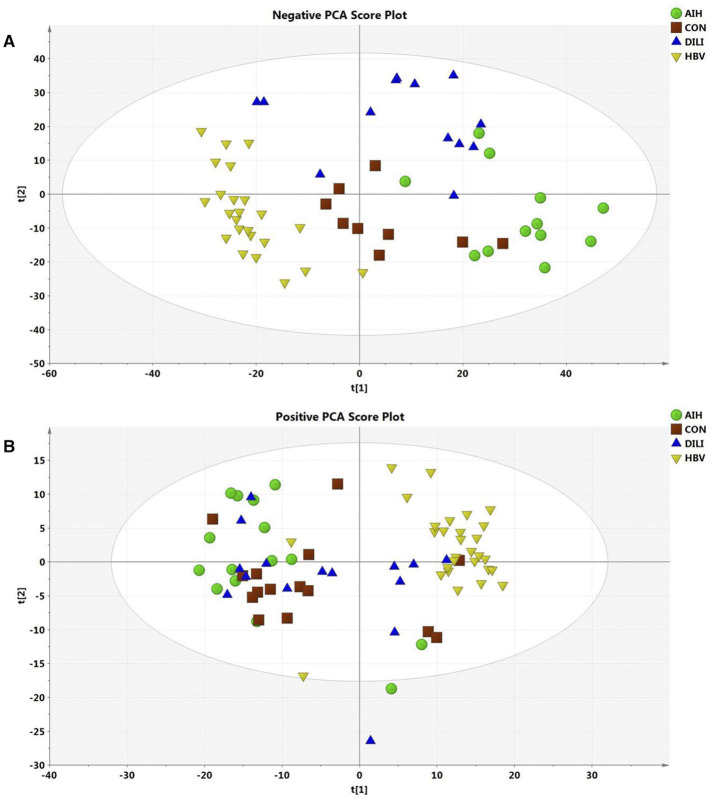
Score plot of PCA model for AIH, CON, DILI, and HBV with the first two principal components. **(A)** The PCA1 and PCA2 explained 27% variation under ESI- mode. **(B)** The PCA1 and PCA2 explained 35% variation under ESI+ mode.

**Figure 2:**
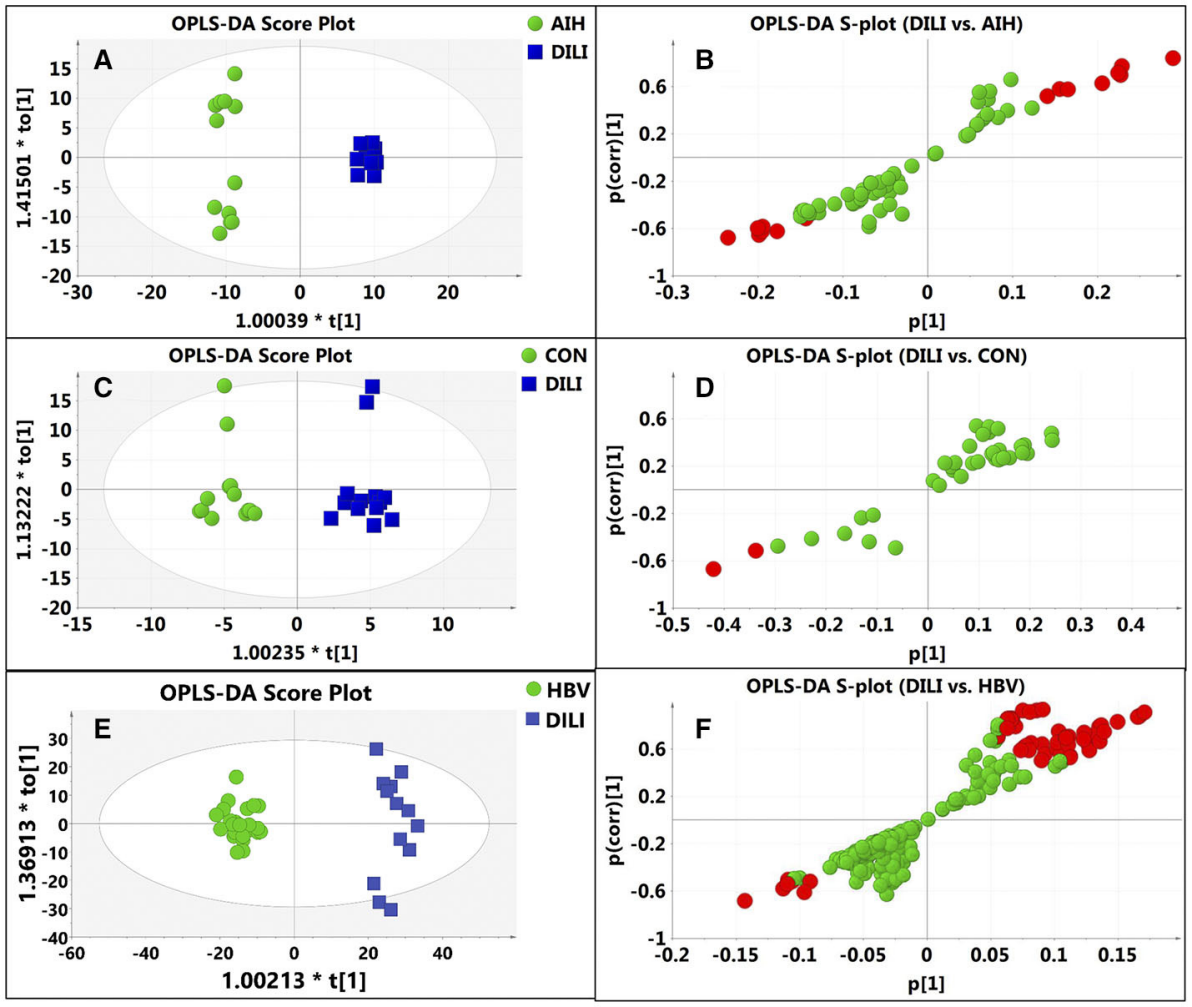
Discrimination of DILI patients from AIH, HBV, and healthy controls according to orthogonal projection to latent structures discriminate analysis (OPLS-DA) model in the ESI- mode. The points in red indicate the identified biomarkers. **(A)** Score plot of the OPLS-DA model for the pair-wise comparisons between the AIH and DILI; **(B)** S-plot of the OPLS-DA model for the AIH and DILI; **(C)** Score plot of the OPLS-DA model for the CON and DILI; **(D)** S-plot of the OPLS-DA model for the CON and DILI; **(E)** Score plot of the OPLS-DA model for the HBV and DILI; **(F)** S-plot of the OPLS-DA model for the HBV and DILI.

**Figure 3:**
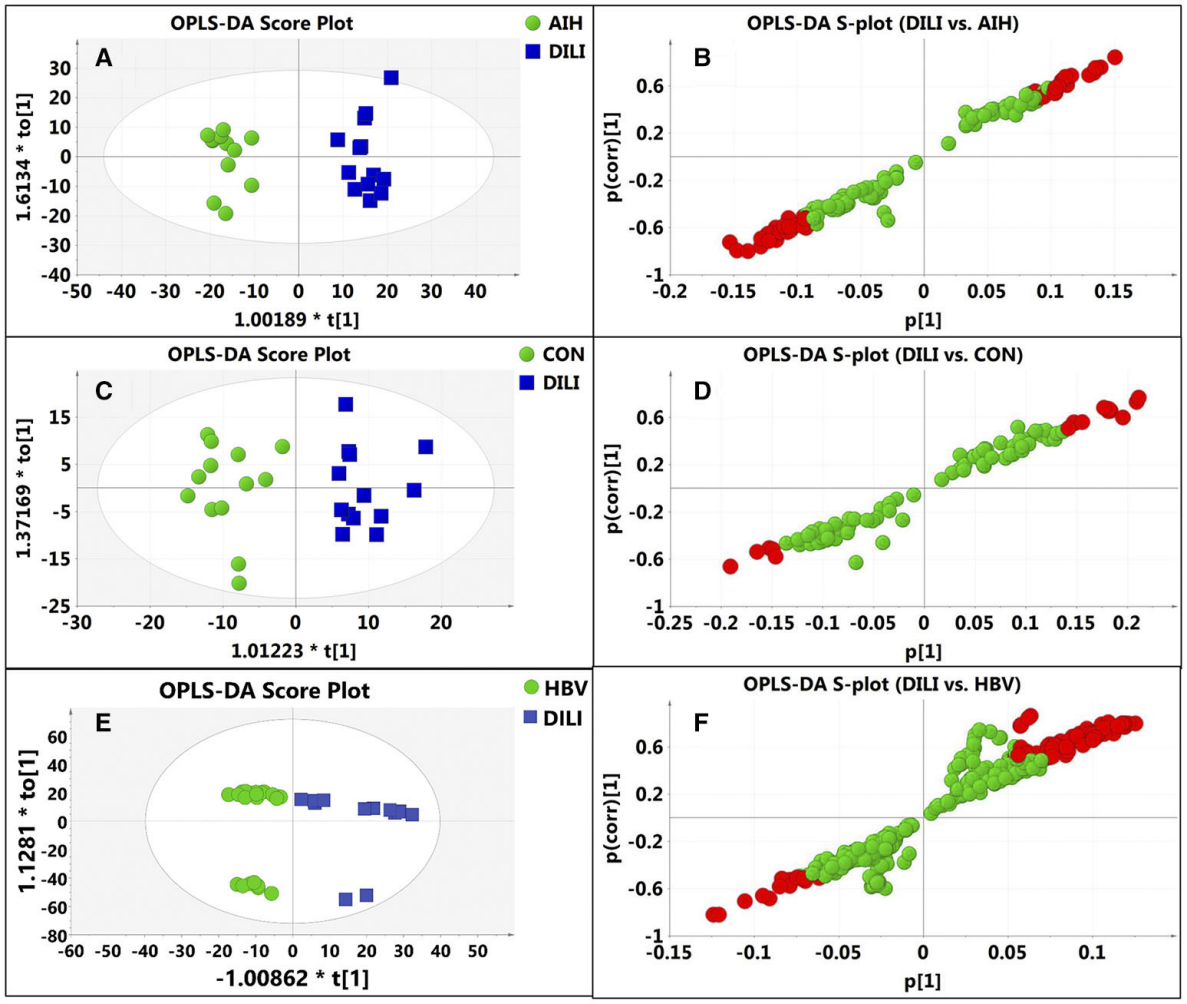
Discrimination of DILI patients from AIH, HBV, and healthy controls according to orthogonal projection to latent structures discriminate analysis (OPLS-DA) model in the ESI+ mode. The points in red indicate the identified biomarkers. **(A)** Score plot of the OPLS-DA model for the pair-wise comparisons between the AIH and DILI; **(B)** S-plot of the OPLS-DA model for the AIH and DILI; **(C)** Score plot of the OPLS-DA model for the CON and DILI; **(D)** S-plot of the OPLS-DA model for the CON and DILI; **(E)** Score plot of the OPLS-DA model for the HBV and DILI; **(F)** S-plot of the OPLS-DA model for the HBV and DILI.

After data normalization, log transformation, and Pareto scaling, 850 metabolites were identified to be significantly different between the PM-DILI and other groups by multivariate analysis. PCA was used as an unsupervised method to examine the overall differences among groups and revealed that the metabolites in PM-DILI were vastly different from the other groups (Figure 1). OPLS-DA (Figures 2, 3) was then used as a supervised method capable of performing classification and discrimination analysis to comprehensively examine the metabolites and revealed significant differences in the paired groups. Based on the criteria of VIP > 1 and a |P(corr)| > 0.5, the 217 molecular-feature ions from the ESI+ and ESI– mode analyses were combined to further identify the molecular formulas. The accurate mass charge ratios of these ions were tentatively identified against the online METLIN database. Finally, a total of 24 potential biomarkers, including 9 metabolites in ESI+ mode and 15 metabolites in ESI– mode, were identified and listed in Table 2.

**Table 2:** Identification of significantly changed metabolites.

**Metabolites name**	**RT (min)**	**Formula**	**Mass^a^ (m/z)**
**Data from the ESI**– **mode**			
4-Cresol	1.20	C7H8O	107.0503
Phenylalanine	1.06	C9H11NO2	164.0729
P-cresol sulfate	1.34	C7H8O4S	187.0042
Oxalosuccinic acid	1.33	C6H6O7	189.0027
D-Glucuronic acid	0.90	C6H10O7	193.0388
Deoxyribose 5-phosphate	3.31	C5H11O7P	213.0217
Melatonin	3.97	C13H16N2O2	231.1098
Inosine	12.66	C10H12N4O5	267.0696
PA(17:2(9Z,12Z)/0:0)^a^	18.70	C20H37O7P	419.2069
(25S)-5β-cholestane-3α,7α,12α,26-tetrol	23.11	C27H48O4	435.3601
Glycochenodeoxycholate	4.33	C26H43NO5	448.2976
LysoPE (0:0/22:6(4Z,7Z,10Z,13Z,16Z,19Z))^b^	13.94	C27H44NO7P	524.2746
Taurocholate	15.00	C26H45NO8S	530.2818
PA(14:1(9Z)/13:0)^c^	19.20	C30H57O8P	575.3758
PG(14:1(9Z)/18:3(6Z,9Z,12Z))^d^	6.53	C38H67O10P	713.4488
CoA	15.02	C21H36N7O16P3S	766.1094
**Data from the ESI+** **mode**			
Valine	1.02	C5H11NO2	118.0875
Methionine	1.05	C5H11NO2S	150.0585
Phenylalanine	1.06	C9H11NO2	166.0875
Coniferyl aldehyde	18.76	C10H10O3	179.0719
Tyrosine	1.05	C9H11NO3	182.0776
Phytosphingosine	11.75	C18H39NO3	318.3045
PE (17:2(9Z,12Z)/0:0)^e^	10.42	C22H42NO7P	464.2905
Bilirubin	19.93	C33H36N4O6	585.2639
DG(17:2(9Z,12Z)/20:0/0:0)^f^	19.93	C40H74O5	635.5741

a *[M-H]^−^ for the ESI– mode and [M+H]^+^ for the ESI+ mode*.

b *Compound in the pathway is LPA*.

c *Compound in the pathway is L-2-LPE*.

d *Compound in the pathway is PA*.

e *Compound in the pathway is PG*.

f *Compound in the pathway is L-1-LPE*.

g *Compound in the pathway is DG*.

**Figure 4:**
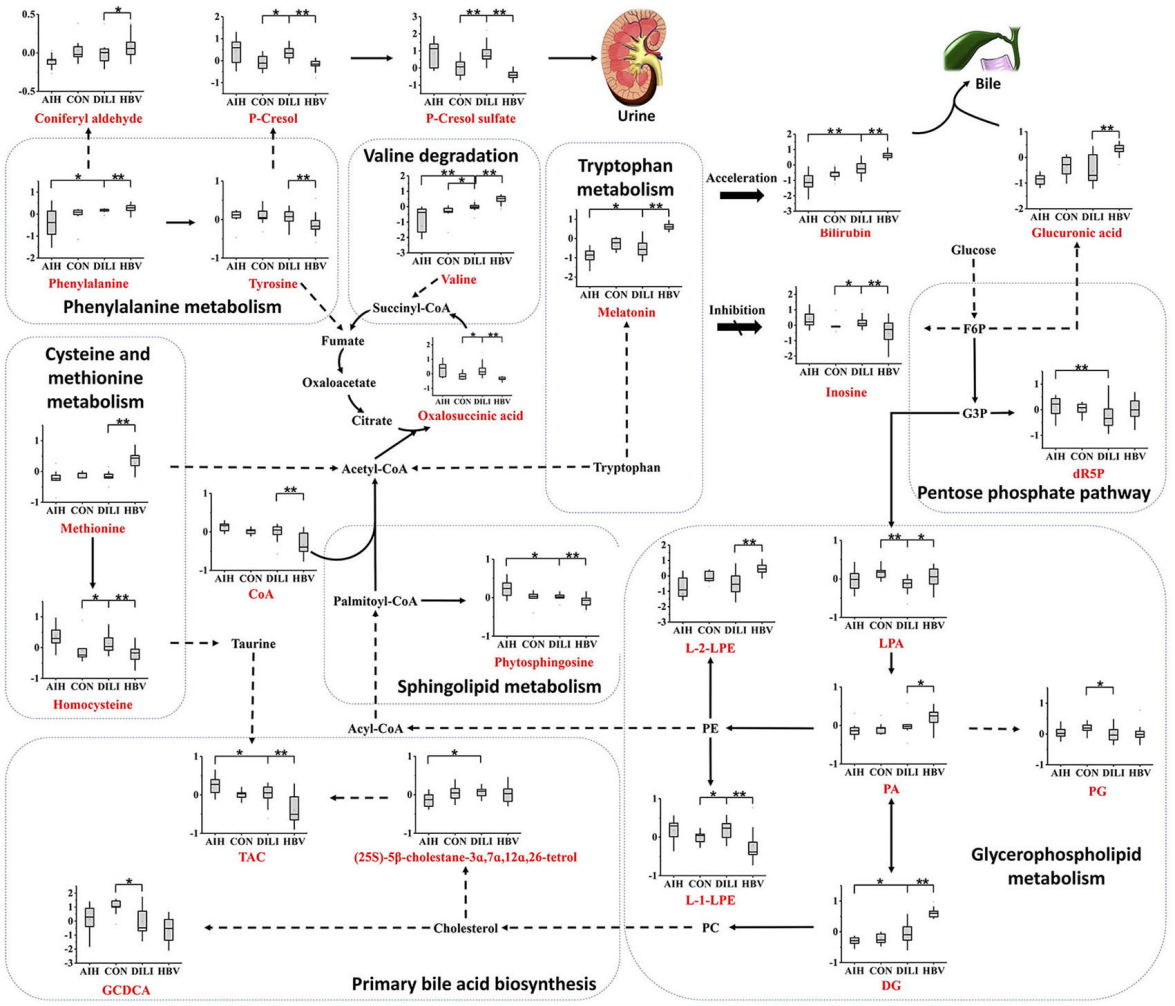
Metabolic network of the significantly changed metabolites. The significantly changed metabolites are shown under the normalized contents. All the *P* values were calculated using Mann-Whitney Test, * and ** represent *p* < 0.05 and *p* < 0.01 compared with the DILI group, respectively. DG, 1,2-diacylglycerol; dR5P, deoxyribose 5-phosphate; PA, phosphatidic acid; PC, phosphatidylcholine; PE, phosphoethanolamine; PG, phosphatidylglycerol; L-1-LPE, L-1-lysophosphoethanolamine; L-2-LPE, L-2-lysophosphoethanolamine; LPA, lysophosphatidic acid; TCA, taurocholic acid; GCDCA, glycochenodeoxycholic acid; F6P, fructose-6-phosphate; G6P, glucose-6-phosphate.

The average normalized quantities of the identified metabolites were orderly clustered and plotted on a heat map based on their Pearson correlation coefficients. The metabolic differences between the PM-DILI and other groups are mapped in Figure 4 according to the KEGG pathways database. We found that the disturbances of some metabolic pathways, such as the metabolisms of three essential amino acids (i.e., tryptophan, valine, phenylalanine), glycerophospholipid metabolism, primary bile acid biosynthesis, and sphingolipid metabolism may specifically contribute to PM-DILI but not to other diseases.

### Decision Tree Learning for PM-DILI Diagnosis

**Figure 5:**
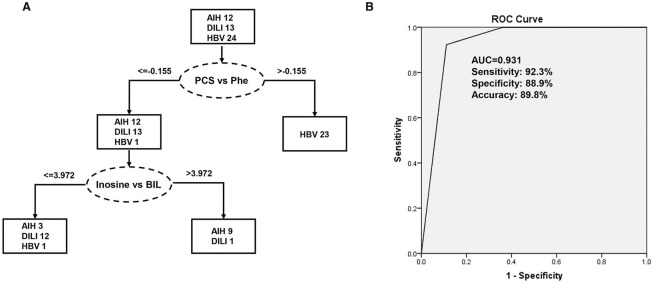
Decision tree classification model for the differential diagnosis of DILI. PCS, P-cresol sulfate; Phe, phenylalanine; BIL, bilirubin. **(A)** Flowchart of the discriminating DILI based on the two ratios of four marker metabolites; **(B)** Results of the ROC tests that correspond to flowchart A.

The 276 randomly combined pairs of 24 identified metabolites were employed to create a decision tree model for the differential diagnosis of the PM-DILI from other diseases using SPSS software. Ultimately, the best two pairs, the ratios of P-cresol sulfate vs. phenylalanine and inosine vs. bilirubin, were selected. Along with a 10-fold cross-validation, the decision tree for PM-DILI prediction achieved sensitivity (positive predictive values) of 92.3%, specificity (negative predictive values) of 88.9%, accuracy of 89.8%, and an error risk of 0.205 ± 0.061. The ROC curve for this decision tree is plotted in Figure 5, and it yielded an AUC of 0.931.

## Discussion

DILI has always been an important cause of ALF, and the diagnosis of DILI remains a challenge. In the present study, plasma metabolomics were employed to investigate the metabolic differences between PM-DILI, HBV, AIH patients, and healthy individuals, to identify a simple and high probability method of diagnosis of PM-DILI.

### Different Metabolic Features of PM-DILI and Relevant Diseases

Based on the results of the current analysis, we found that a few essential amino acids and glucose metabolites play important roles in the differentiation of PM-DILI from other symptomatically similar diseases and healthy states. The consumption of glucose leads to increased glucuronic acid volume through the pentose and glucuronate interconversion pathway in HBV and increased 2-Deoxy-D-ribose-5P (dR5P) volume through the pentose phosphate pathway in AIH. It is well known that glucuronic acid conjugates with xenobiotics, such as drugs and bilirubin, with a high probability of making them more water-soluble and eliminating them from the body through the urine or bile (15). In the present study, the significantly elevated levels of D-glucuronic acid observed in the HBV patients might induce impairment in liver detoxification. The pentose phosphate pathway is the primary pathway for the generation of nicotinamide adenine dinucleotide phosphate (NADPH), which is beneficial in the prevention of oxidative stress (16–18). It can be deduced that AIH patients endure more oxidative stress than PM-DILI patients. Considering that the metabolites of the TCA cycle and LysoPE were elevated in PM-DILI and AIH patients, glycerophospholipid metabolism might have been accelerated by the degradation of phosphoethanolamine (PE) to provide adequate energy in these patients.

In addition to glucose metabolism, changes in plasma AA concentrations may also have influenced various biological functions by impairing hepatic function and subsequently caused hyperinsulinemia and hyperglucagonemia (19). For example, methionine (Met) was upregulated in HBV and decreased in AIH and PM-DILI, and the change of Met is frequently associated with the change of related metabolites, such as taurine and cysteine (20). Taurocholic acid is a conjugate of taurine and cholic acid and was observed in our study, which contrasts with the changes in Met. Furthermore, a stepwise increase in the plasma levels of valine was observed from AIH to PM-DILI to HBV. Valine is one of the branched-chain amino acids that have a stimulatory effect on glutamine synthesis and is a major source of nitrogen (21–23). Given that the liver plays a role in the regulation of the glutamine synthesis (24), plasma levels of valine may reflect changes in hepatic function. Additionally, γ- glutamyl dipeptides, the biomarkers for many liver diseases (8), are also synthesized from glutamate, which suggests that valine has the potential to discriminate PM-DILI from other liver diseases.

Furthermore, a few metabolites of amino acids observed in this study were also able to differentiate PM-DILI from other diseases. For example, melatonin is an indolamine product of tryptophan and is involved in the regulation of circadian rhythms. Melatonin also possesses an ability to modulate numerous molecular pathways, including those related to cellular injury (25), oxidative stress (26–28), and inflammation (29–32). The plasma melatonin is altered in different diseases and pathological states; i.e., decreased levels are associated with the hyperactivation of the immune system (33), and elevated levels are correlated with the severity of cirrhosis and hepatic encephalopathy (34). In our study, melatonin were reduced in AIH group and elevated in HBV group compared to the PM-DILI and healthy group. It can be concluded that the levels of melatonin in AIH may be downregulated by abnormal autoimmune reactions against hepatocytes and the liver injuries of HBV patients could be more serious than those of the PM-DILI patients.

Moreover, sphingolipid metabolism was abnormal in the AIH and HBV patients compared to the PM-DILI patients. Sphingolipids have been reported to play important roles in mediating many biological functions, such as cell growth, apoptosis, senescence, and differentiation (35–38). Phytosphingosine is structurally similar to sphingosine and is a precursor of ceramide, which is also regarded as an important cellular signal for inducing apoptosis. Phytosphingosine was downregulated in the HBV patients and upregulated in the AIH patients, but no significant difference was observed between the PM-DILI patients and healthy controls. Thus, it could be inferred that apoptosis was not obvious in PM-DILI patients.

### Diagnostic Potentials of the Differential Metabolites

The underlying mechanisms of DILI induced by distinctive drugs may also be different. However, the changes in some metabolites in the plasma might be similar among the DILI samples (8). In this study, all the metabolites with significant changes were regarded as candidate biomarkers, whose diagnostic potentials need to be investigated. In order to precisely differentiate DILI (PM-DILI) cases from cases with other liver diseases, a decision tree analysis that can be used in clinical settings was implemented. Considering the error of each detection, we performed the decision tree analysis with the ratios of identified metabolites. The results indicated that the ratios of P-cresol sulfate vs. phenylalanine and inosine vs. bilirubin need to be investigated for their potential utility in the differential diagnosis of DILI.

## Conclusion

In summary, our LC–HRMS-based metabolic profiling analysis of plasma samples provides an integrated view of the metabolic features of PM induced DILI. The differential metabolites between DILI and relevant liver diseases were screened and identified, and the results indicated that the metabolic alterations in DILI were mainly related to amino acid and sphingolipid metabolisms. These findings can potentially provide valuable information for the diagnoses of DILI. The diagnostic potentials of the differential metabolites found in the plasma samples revealed that the ratios of P-cresol sulfate vs. phenylalanine and inosine vs. bilirubin exhibited good sensitivity and specificity in differentiating DILI from AIH and HBV. Hence, these factors have great potential as biomarkers of DILI in clinical diagnosis.

## Data Availability Statement

The raw data supporting the conclusions of this article will be made available by the authors, without undue reservation.

## Ethics Statement

The studies involving human participants were reviewed and approved by Medical Ethics Committee of Fifth Medical Center of Chinese PLA General Hospital. The patients/participants provided their written informed consent to participate in this study. Written informed consent was obtained from the individual(s) for the publication of any potentially identifiable images or data included in this article.

## Author Contributions

YH and MN: LC-MS data analysis and manuscript preparation. YH: sample collection and preparation. MN, J-bW, and X-hX: experimental design. XZ, Z-tZ, S-sC, S-sL, ZS, JJ, AH, Y-mG, Z-fB, and Z-sZ: helped in execution of research. All authors contributed to the article and approved the submitted version.

## Conflict of Interest

The authors declare that the research was conducted in the absence of any commercial or financial relationships that could be construed as a potential conflict of interest.
